# Editorial: Safety and efficacy of stents and flow diverters used for embolization of acutely-ruptured intracranial aneurysms in the acute stage

**DOI:** 10.3389/fneur.2023.1258677

**Published:** 2023-08-14

**Authors:** Bulang Gao, Hongqi Zhang, Xianli Lv, Robert P. Ostrowski, Conghui Li

**Affiliations:** ^1^Department of Medical Research, Shijiazhuang People's Hospital, Hebei Medical University, Shijiazhuang, China; ^2^Department of Neurosurgery, Xuanwu Hospital of Capital Medical University, Beijing, China; ^3^Department of Neurosurgery, Beijing Tsinghua Changgung Hospital, School of Clinical Medicine, Tsinghua University, Beijing, China; ^4^Department of Experimental and Clinical Neuropathology, Mossakowski Medical Research Centre, Polish Academy of Sciences, Warsaw, Poland; ^5^Department of Neurosurgery, Hebei Medical University First Hospital, Shijiazhuang, Hebei, China

**Keywords:** stents, flow diverters, acutely ruptured, intracranial aneurysms, endovascular treatment

Currently, the treatment of acutely-ruptured intracranial aneurysms (ARIAs) using stents or flow diverters for endovascular embolization is controversial. Some authors favor the use of stents and flow diverters, but others do not. For one thing, the standardized embolization procedure has not been established for the use of stents or flow diverters. For another, the use of stents or flow diverters may cause additional adverse events in the acute phase of aneurysm rupture compared with coils only. Nonetheless, because ARIAs may have unfavorable morphology for endovascular embolization, the use of stents or flow diverters is necessary.

In different countries and regions across the world, the development of skills using stents or flow diverters for ARIAs is not balanced. In China with a large population and a great number of aneurysm patients, endovascular treatment with stents or flow diverters is necessary because of the micro-invasiveness and fast recovery, and a great deal of experience using the stents or flow diverters has been accumulated. With accumulation of such experience, standardized embolization procedures should be established to improve the safety and efficacy of stents or flow diverters for ARIAs. Treatment of ARIAs in the acute phase is critical to the prognosis of these patients, and timely treatment is able to prevent a secondary rupture and will promote further aggressive treatment, beneficial to the recovery.

In this Research Topic of 10 articles, the aim is to bring together the latest quality articles and provide an up-to-date and comprehensive overview of the latest research hotspots from researchers for the treatment of ARIAs using intracranial stents and flow diverters with or without adjunctive coiling. In particular, the following specific themes have been touched upon: periprocedural complication rates, re-rupture of ARIAs, angiographic and clinical outcomes, and ischemic events when using intracranial stents and flow diverters for ARIAs. Four articles focused on traditional intracranial stents in assisting coiling of ARIAs (Liu et al.; Wu et al.; Zhang, Wu et al.; Zhang, Zhang et al.), one meta-analysis on staged stenting of intracranial stents and Pipeline embolization device (PFD) for wide-necked ARIAs (Wei et al.), two articles on the PED treatment of intracranial aneurysms (Li et al.; You et al.), one meta-analysis of efficacy and safety on the use of the Willis covered stent in the treatment of blood blister-like aneurysm (Tan et al.), one bibliometric study of worldwide productivity and research trends of publications concerning stent application in ARIAs (Chen et al.), and one on automatic risk prediction of intracranial aneurysm on CTA image with convolutional neural networks and radiomics analysis (Xie et al.).

In the bibliometric study of worldwide productivity and research trends of publications concerning stent application for ARIAs (Chen et al.), 275 publications were included, the research focus was ARIAs and application of stents during interventional procedures, and the main trends of research were development of materials and safety of stent application in ARIAs.

In the study of stent-assisted coiling vs. coiling alone for tiny ARIAs (Zhang, Wu et al.), it was found that stent-assisted coiling may increase the incidence of hemorrhagic events with favorable angiographic outcomes and comparable clinical outcomes as compared with stand-alone coiling and that the low-profile visualized intraluminal support (LVIS) stent may improve the safety compared with the lazer-cut stent. Two other studies (Liu et al.; Wu et al.) confirmed the effect of the LVIS stent for ARIAs with favorable angiographic and clinical outcomes. In one study with 41 patients with ARIAs (Liu et al.), the complete aneurysm occlusion rate was 70.7% immediately after embolization and 83.3% at 13.9-month angiographic follow-up, the favorable clinical outcome rate at follow-up was 92.7%, and intraoperative thrombosis and hemorrhage occurred in two (4.9%) and one (2.4%) patients, respectively. In the other study with a LVIS stent being deployed within an Enterprise stent for the treatment of 30 patients with 34 acutely-ruptured intracranial vertebrobvasilar artery-dissecting aneurysms (Wu et al.), all aneurysms were successfully treated in the acute stage, six patients (20.0%) experienced severe in-hospital adverse events (two deaths, 6.7%), aneurysm rebleeding occurred in one patient (3.3%), and three ischemic events happened. At 12-month follow-up, the complete aneurysm occlusion rate was 93.3%, and the incidence of dependence of death (mRS score of 3–6) at discharge and at the last follow-up was 16.7 and 14.3%, respectively.

Two studies (Zhang, Zhang et al.; Wei et al.) investigated staged stenting for ARIAs with initial coiling followed by scheduled delayed stenting, resulting in comparable or better angiographic complete occlusion rates, procedure-related complication rate, and clinical outcomes at follow-up. In one study (Zhang, Zhang et al.), the propensity score-matched method was used to balance the data in the staged stenting arm and the conventional stent-assisted coiling arm, without using the flow diverters, and comparable clinical, angiographic, and procedure-related complication rates were obtained in both arms. In the other study of a meta-analysis and systematic review including both conventional intracranial stents and flow diverters in 5 studies with 143 patients with ARIAs (Wei et al.), a high aneurysm occlusion rate, favorable clinical outcomes and lower procedure-related complication rates have been achieved in the staged stenting group.

Two articles investigated the effect of the PED vs. traditional coils in embolization of intracranial aneurysms (Li et al.) or the incidence and prediction of in-stent stenosis after PED deployment for intracranial aneurysms treatment (You et al.). In one study of a meta-analysis with 10 studies and 1,400 patients enrolled (Li et al.), the PED had higher rates of complete aneurysm occlusion but lower rates of aneurysm retreatment in comparison with traditional coils, but traditional coils was superior to the PED group in terms of procedure-related intracranial hemorrhage and other procedure-related complications, and favorable functional outcome (mRS ≤ 2). In the other study with 240 patients and 252 aneurysms (You et al.), it was found that in-stent stenosis is a common angiographic finding after PED implantation for intracranial aneurysms and is presented as a largely benign course through long-term follow-up and that younger patients and longer procedure durations were risk factors for developing in-stent stenosis.

A meta-analysis of efficacy and safety of the Willis covered stent for treating blood blister-like aneurysms including eight studies and 104 patients (Tan et al.) found that the Willis covered stent could be effectively and safely applied for the treatment of this kind of aneurysms. In the last article exploring automatic risk prediction of intracranial aneurysm on CTA image with convolutional neural networks (CNN) and radiomics analysis (Xie et al.), the incorporation of CNN and radiomics analysis can improve the prediction performance, and the selected optimal feature set can provide essential biomarkers for the determination of rupture risk.

In conclusion, this Research Topic provides an up-to-date and comprehensive overview of the latest research hotspots regarding the use of intracranial stents and flow diverters for the treatment of intracranial aneurysms and it is a great step forward even though not all fields have been covered.

## Author contributions

BG: Conceptualization, Formal analysis, Investigation, Methodology, Validation, Visualization, Writing—original draft, Writing—review and editing. HZ: Conceptualization, Resources, Validation, Visualization, Writing—review and editing. XL: Conceptualization, Formal analysis, Validation, Visualization, Writing—review and editing. RO: Conceptualization, Validation, Visualization, Writing—review and editing. CL: Conceptualization, Validation, Visualization, Writing—review and editing.

